# Different macrophage polarization between drug-susceptible and multidrug-resistant pulmonary tuberculosis

**DOI:** 10.1186/s12879-020-4802-9

**Published:** 2020-01-29

**Authors:** Hyun Jin Cho, Yun-Ji Lim, Jhingook Kim, Won-Jung Koh, Chang-Hwa Song, Min-Woong Kang

**Affiliations:** 1 Department of Thoracic and Cardiovascular Surgery, Chungnam National University Hospital, Chungnam National University College of Medicine, 282 Munhwa-ro, Jung-gu, Daejeon, 35015 South Korea; 2 Research Institute for Medical Sciences, Chungnam National University College of Medicine, Daejeon, South Korea; 3 Department of Microbiology, Chungnam National University College of Medicine, 266 Munhwa-ro, Jung-gu, Daejeon, 35015 South Korea; 4 Department of Thoracic and Cardiovascular Surgery, Samsung Medical Center, Sungkyunkwan University School of Medicine, Seoul, South Korea; 5 Division of Pulmonary and Critical Care Medicine, Department of Medicine, Samsung Medical Center, Sungkyunkwan University School of Medicine, Seoul, South Korea

**Keywords:** Tuberculosis, Macrophages, Macrophage polarization, Multidrug-resistant

## Abstract

**Background:**

Macrophages play a key role in the infection process, and alternatively activated macrophages (M2 polarization) play important roles in persistent infection via the immune escape of pathogens. This suggests that immune escape of pathogens from host immunity is an important factor to consider in treatment failure and multidrug-resistant tuberculosis (MDR-TB)/extensively drug-resistant tuberculosis (XDR-TB). In this study, we investigated the association between macrophage polarization and MDR-TB/XDR-TB and the association between macrophage polarization and the anti-TB drugs used.

**Methods:**

iNOS and arginase-1, a surface marker of polarized macrophages, were quantified by immunohistochemical staining and imaging analysis of lung tissues of patients who underwent surgical treatment for pulmonary TB. Drug susceptibility/resistance and the type and timing of anti-tuberculosis drugs used were investigated.

**Results:**

The M2-like polarization rate and the ratio of the M2-like polarization rate to the M1-like polarization rate were significantly higher in the MDR-TB/XDR-TB group than in the DS-TB group. The association between a high M2-like polarization rate and MDR-TB/XDR-TB was more pronounced in patients with a low M1-like polarization rate. Younger age and a higher M2-like polarization rate were independent associated factors for MDR-TB/XDR-TB. The M2-like polarization rate was significantly higher in patients who received anti-TB drugs containing pyrazinamide continuously for 4 or 6 weeks than in those who received anti-TB drugs not containing pyrazinamide.

**Conclusions:**

The M2-like polarization of macrophages is associated with MDR-TB/XDR-TB and anti-TB drug regimens including pyrazinamide or a combination of pyrazinamide, prothionamide and cycloserine.

## Background

Tuberculosis (TB) remains one of the top 10 causes of death worldwide, and the emergence of strains resistant to anti-TB drugs threatens TB control [1, 2]. Multidrug-resistant TB (MDR-TB) is TB that is resistant to at least isoniazid and rifampicin, and extensively drug-resistant TB (XDR-TB) is defined as MDR-TB that is resistant to fluoroquinolone and second-line injectable drugs [1]. In 2016, an estimated 490,000 people newly developed MDR-TB worldwide, and the MDR-TB burden is increasing [1, 2]. Only 1 in 5 people who needed treatment for MDR-TB actually received it, and 54% of those who started treatment for MDR-TB were cured in the 2014 cohort [1].

A tuberculous granuloma, which is a characteristic pathological hallmark of TB, is an organized collection of macrophages, lymphocytes, and multinucleate giant cells that is a product of the interaction between *Mycobacterium tuberculosis* (*Mtb*) and the host immune system [3, 4]. Macrophages play a key role in the formation of tuberculous granulomas and the infection process, as the immune cells responsible for the first-line defense against *Mycobacterium* infection [3, 5–7]. Activated macrophages are polarized into two different phenotypes and perform two distinct roles in the immune system. Classically activated macrophages (M1 polarization) mediate inflammatory responses and increase the microbicidal and tumoricidal capacity [8]. In contrast, alternatively activated macrophages (M2 polarization) play important roles in tissue repair, tumor progression, and persistent infection via the immune escape of tumors and pathogens [4, 9, 10]. This suggests that immune escape of pathogens from the host immunity is an important factor to consider in treatment failure and MDR-TB/XDR-TB. Previously, we found that alternatively activated macrophages were more abundant in the lung tissue of MDR-TB patients than in the lung tissue of new-onset TB patients, although the sample size was small [11].

The treatment of TB requires long-term drug administration, especially for MDR-TB/XDR-TB. The prolonged use of anti-TB drugs is essential for eradication of *Mtb* but may also affect host defense systems, and the drugs may cause complications directly. Some antibiotics have immunomodulatory properties in vitro [12]. Rifampicin exerts anti-inflammatory effects via the suppression of nuclear factor-kappa B in neurodegenerative diseases [13, 14]. Pyrazinamide treatment can influence the host immune response by reducing pro-inflammatory cytokine production in *Mtb* infection [15]. First-line anti-TB treatment in patients with tuberculous pleuritis induced M2 polarization of pleural macrophages [16]. Moxifloxacin suppresses the production of pro-inflammatory cytokines [17]. These observations suggest that anti-TB drugs can modulate the host immune response.

We hypothesized that MDR-TB/XDR-TB has a positive association with M2-like polarization in the tissue microenvironment and that the type of anti-TB drugs used before surgery is associated with the M2 polarized environment. This study investigated the dominant macrophage polarization in tuberculous granulomas obtained from surgically resected lung specimens of MDR-TB/XDR-TB and drug-susceptible TB (DS-TB) patients, and analyzed which anti-TB drugs are correlated with the M2-like polarized environment in MDR-TB/XDR-TB patients.

## Methods

### Study population and tissue specimens

All patients who underwent surgical treatment for pulmonary TB at two centers (Chungnam National University Hospital, Daejeon, South Korea and Samsung Medical Center, Seoul, South Korea) between January 1998 and December 2014 were identified through the patient data registry of each hospital, and their medical records were reviewed retrospectively. The institutional review boards of both institutions approved this study (IRB No. CNUH 2015–10–032-002; SMC 2015–09–063-001), and waived the need for informed consent. Tissues for staining were obtained from tuberculous granulomas of specimens and subjected to immunohistochemical staining and imaging analysis for iNOS and Arginase-1, a surface marker of polarized macrophages, by two researchers who were blinded to the clinical data. The type and timing of the anti-TB drugs used and data on drug-sensitivity tests (DSTs) were obtained from the data registry of each hospital and retrospectively reviewed medical records. One hundred twenty-five patients underwent surgical resection for MDR/XDR-TB or DS-TB. Three patients without sufficient tissue for staining were excluded. Two sections from a specimen from each patient were obtained, and one section each was stained with iNOS/CD68 and Arginase-1/CD68.

### Immunohistochemistry

Alveolar macrophages were identified by CD68 surface markers, and M1 or M2 macrophages were distinguished using iNOS and arginase-1 surface markers, respectively. Paraffin-embedded lung tissue sections (4 μm) of the TB patients were pretreated with ethylenediaminetetraacetic acid (EDTA) antigen-retrieval citrate buffer (C9999; Sigma Aldrich) at 120 °C for 4 min in a pressure boiler. The sections were incubated with anti-CD68 (sc-20,060, 1:100; Santa Cruz Biotechnology), anti-iNOS (sc-651, 1:100; Santa Cruz Biotechnology) and anti-arginase-1 (sc-20,150, 1:100; Santa Cruz Biotechnology) primary antibodies overnight at 4 °C. Immunoreactivity was detected using Alexa Fluor 488-conjugated goat anti-mouse IgG secondary antibody (A-11001, 1:200; Thermo) and Alexa Fluor 594-conjugated goat anti-rabbit IgG secondary antibody (A-11012, 1:200; Thermo). The slides were then mounted.

### Imaging and quantitative analysis

Next, the slides were examined using confocal microscopy (400× field). All images were collected using laser excitation at 488 and 594 nm. The contrast/brightness was adjusted, and the images were laid out using Adobe Photoshop CS6 (Adobe Systems, Inc.). The average numbers of iNOS^+^ or Arginase-1^+^ cells or CD68^+^ cells were quantified in each section using ImageJ software (NIH). The M1 or M2 polarization rate (%) was defined as (iNOS^+^ cell count / CD68^+^ cell count) × 100 and (Arginase-1^+^ cell count / CD68^+^ cell count) × 100, respectively.

### Statistical analysis

All statistical analyses were performed using SPSS® software (ver. 19.0; IBM Corp., Armonk, NY, USA). Categorical variables were compared between groups using Pearson’s chi-square test or Fisher’s exact test, continuous variables were compared using Student’s *t*-test, and the Mann–Whitney *U*-test was used when the data were not normally distributed. Correlations between continuous variables were estimated using partial correlation analysis. A logistic regression model was used for univariate and multivariate analyses of covariate risk factors. Covariates with a *P*-value < 0.1 in the univariate analyses were included in the multivariate analysis. The results are expressed as odds ratios (ORs) with 95% confidence intervals (CIs). *P*-values < 0.05 were considered statistically significant.

## Results

### Patient characteristics

This study enrolled 122 patients [mean age, 38.1 ± 11.9 years; 69 males (56.6%)]. The MDR-TB and XDR-TB groups had 65 patients and 33 patients, respectively, and the DS-TB group comprised 24 patients (19.7%); their characteristics are compared in Table 1. The DS-TB group was significantly older than the MDR-TB/XDR-TB group. Most patients in the MDR-TB/XDR-TB group underwent surgery because of disease progression or severe complications during the long-term use of second-line anti-TB drugs. One patient was operated on without using second-line anti-TB drugs, and 97 patients were operated on during second-line anti-TB drug use. The patients in the DS-TB group underwent surgery to evaluate mass-like lesions in the lung parenchyma. Eight patients (33.3%) empirically received first-line anti-TB drugs before surgery, and 16 patients (66.7%) started taking first-line anti-TB drugs after the identification of TB granuloma from resected tissue.

**Table 1:** Clinical characteristics of the patients

Characteristic	MDR-TB/XDR-TB (*n* = 98)	DS-TB (*n* = 24)	*P* value
Male sex, n (%)	52 (53.1)	17 (70.8)	0.115
Age, mean ± SD, yr	35.4 ± 10.1	48.9 ± 12.6	< 0.001
Use of anti-TB drugs before surgery, n (%)			
Initial surgery without anti-TB drugs	0 (0)	16 (66.7)	
First-line → surgery	1 (1.1)^a^	8 (33.3)	
Second-line → surgery	97 (98.9)	0 (0)	
Drug-sensitivity test, n (%)	98 (100)	24 (100)	
All sensitive	0 (0)	24 (100)	
Multidrug-resistant	65 (66.3)	0 (0)	
Extensively drug-resistant	33 (33.7)	0 (0)	

*MDR-TB* Multidrug-resistant tuberculosis, *XDR-TB* Extensively drug-resistant tuberculosis, *DS-TB* Drug-susceptible tuberculosis, *SD* Standard deviation

^a^ This patient was operated on without using second-line drugs after multidrug-resistant tuberculosis was diagnosed following long-term use of isoniazid, rifampicin, and ethambutol for relapsed tuberculosis

### Comparison of macrophage polarization between the DS-TB and MDR-TB/XDR-TB groups

**Fig. 1:**
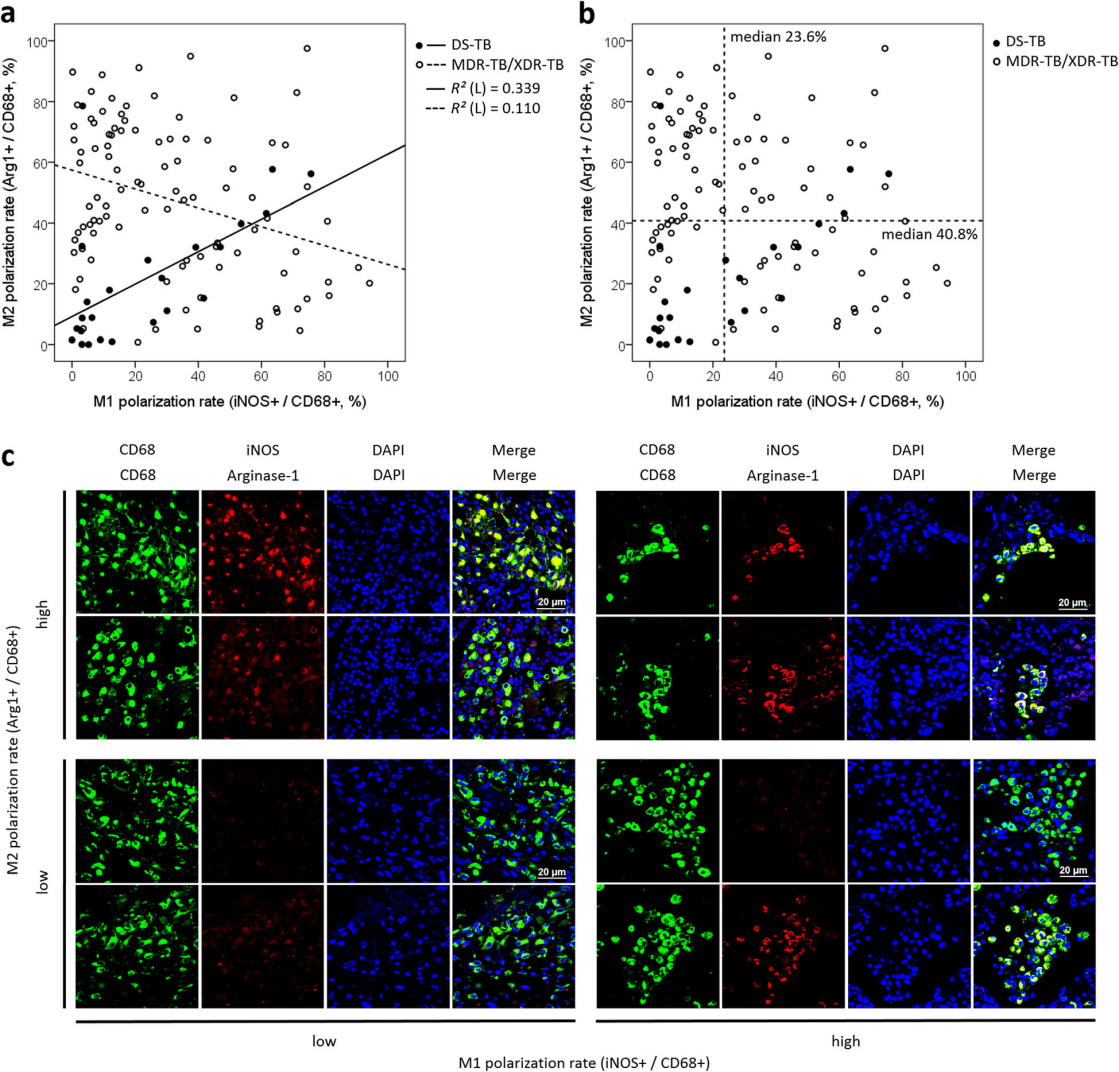
The distribution and correlation of the M1-like and M2-like polarization rates between MDR-TB/XDR-TB and DS-TB. **a** Scatterplot of the M1-like and M2-like polarization rates for each group. In the DS-TB group, the M1-like and M2-like polarization rates had a moderate positive correlation. In the MDR-TB/XDR-TB group, the M1-like and M2-like polarization rates had a moderate negative correlation. ‘r’ is the partial correlation coefficient adjusted by age. **b** The median values of M1-like and M2-like polarization rates were obtained, and 83.3% of the DS-TB group were distributed below the median value of the M2-like polarization rate. **c** The confocal microscopy image (400x field) showed a distinct polarization difference in each quadrant according to the distribution of the M1-like and M2-like polarization rates. All images were obtained with laser excitation at 488 and 594 nm. The iNOS and arginase-1 stains were quantified as the percentage in CD68-positive alveolar macrophages. The average number of iNOS^+^ or Arginase-1^+^ cells / CD68^+^ cells was quantified for each section using ImageJ software (NIH). DS-TB = drug-susceptible tuberculosis; MDR-TB = multidrug-resistant tuberculosis; XDR-TB = extensively drug-resistant tuberculosis; Arg1 = Arginase-1

**Fig. 2:**
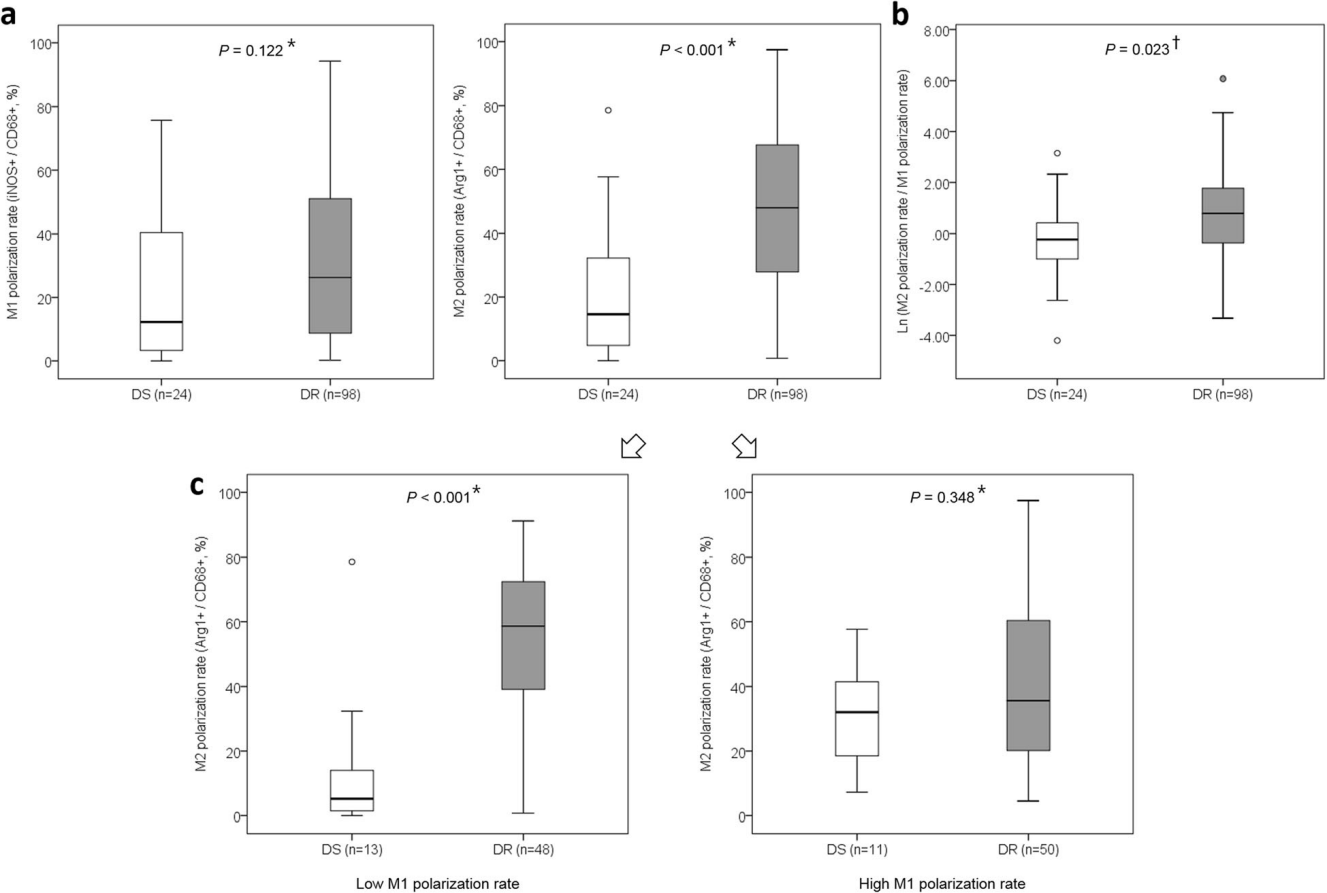
The M1-like and M2-like polarization rates between the DS-TB and MDR-TB/XDR-TB groups. **a** The M1-like polarization rate did not differ significantly between the two groups, while the M2-like polarization rate was significantly higher in the MDR-TB/XDR-TB group than in the DS-TB group. **b** The ratio of the M2-like polarization rate to the M1-like polarization rate (macrophage polarization ratio (MP ratio) = M2-like polarization rate / M1-like polarization rate) was calculated, and the natural logarithm of the ratio [*Ln* (MP ratio)] was compared between the two groups. The *Ln* (MP ratio) was significantly higher in the MDR-TB/XDR-TB group than in the DS-TB group. **c** The M1 polarization rate was dichotomized into the low M1 group and high M1 group based on the median value, and the differences in the M2-like polarization rate between the DS-TB and MDR-TB/XDR-TB groups were analyzed in each M1 group. The M2-like polarization rate of the MDR-TB/XDR-TB group was significantly higher than that of the DS-TB group within the low M1 group, but the M2-like polarization rate was not significantly different between the two groups within the high M1 group. Statistical comparisons between the DS-TB and MDR-TB/XDR-TB groups were performed using Student’s *t*-test (^*^) and the Mann-Whitney *U* test (^†^). DS = dru-susceptible tuberculosis; DR = drug-resistant tuberculosis (MDR-TB/XDR-TB); MDR-TB = multidrug-resistant tuberculosis; XDR-TB = extensively drug-resistant tuberculosis; Arg1 = Arginase-1

The distribution of the M1-like and M2-like polarization rates showed a moderate positive correlation (*R* = 0.582, *P* = 0.004) in the DS-TB group but a moderate negative correlation (*R* = − 0.332, *P* = 0.001) in the MDR-TB/XDR-TB group (*R* = partial correlation coefficient adjusted by age; Fig. 1a). Overall, the median values of M1-like and M2-like polarization rates were 23.6% [IQR, 6.9–47.4%] and 40.8% [IQR, 20.4–66.5%], respectively, and 83.3% of the DS-TB group (20/24 cases) were distributed below the median value of the M2 polarization rate (Fig. 1b). The confocal microscopy image showed a distinct polarization difference in each quadrant according to the distribution of the M1-like and M2-like polarization rates (Fig. 1c). Comparing the macrophage polarization between the DS-TB and MDR-TB/XDR-TB groups, the M2-like polarization rate was significantly higher in the MDR-TB/XDR-TB group than in the DS-TB group (47.9% [IQR, 27.8–67.9%] vs. 14.6% [IQR, 4.6–32.3%], *P* < 0.001), while the M1-like polarization rate did not differ significantly between the two groups (Fig. 2a). The ratio of the M2-like polarization rate to the M1-like polarization rate (macrophage polarization ratio (MP ratio) = M2-like polarization rate / M1-like polarization rate) was calculated, and the natural logarithm of the ratio [*Ln* (MP ratio)] was compared between the two groups. *Ln* (MP ratio) was significantly higher in the MDR-TB/XDR-TB group than in the DS-TB group (0.75 ± 1.72 vs. -0.17 ± 1.56, *P* = 0.023; Fig. 2b). The M1-like polarization rate was dichotomized into a low M1 group and a high M1 group based on the median value (23.6%), and the differences in the M2-like polarization rate between the DS-TB and MDR-TB/XDR-TB groups were analyzed in each M1 group. The M2-like polarization rate of the MDR-TB/XDR-TB group was significantly higher than that of the DS-TB group in the low M1 group (58.6% [IQR, 27.2–72.7%] vs. 5.2% [IQR, 1.2–15.9%], *P* < 0.001), but the M2-like polarization rate was not significantly different between the two groups within the high M1 group (35.6% [IQR, 19.1–61.7%] vs. 32.0% [IQR, 15.2–43.2%], *P* = 0.348; Fig. 2c).

In the univariate and multivariate analyses of factors associated with MDR-TB/XDR-TB, younger age and a higher M2-like polarization rate were independent associated factors for MDR-TB/XDR-TB (Table 2).

**Table 2:** Univariate and multivariate analysis of factors associated with MDR-TB/XDR-TB

Characteristics	Univariate*			Multivariate		
	OR	95% CI	*P* value	OR	95% CI	*P* value
Male sex	0.465	0.177–1.222	0.121	NA	NA	NA
Age	0.897	0.853–0.942	< 0.001	0.901	0.853–0.952	< 0.001
M1-like polarization rate	1.014	0.994–1.034	0.164	NA	NA	NA
M2-like polarization rate	1.050	1.025–1.075	< 0.001	1.047	1.019–1.075	0.001

*MDR-TB* Multidrug-resistant tuberculosis, *XDR-TB* Extensively drug-resistant tuberculosis, *OR* Odds ratio, *CI* Confidence interval, *NA* Not adjusted

* Covariates with a *P*-value < 0.1 in the univariate analysis were included in the multivariate analysis

### Anti-TB drugs and macrophage polarization in the MDR-TB/XDR-TB group

**Fig. 3:**
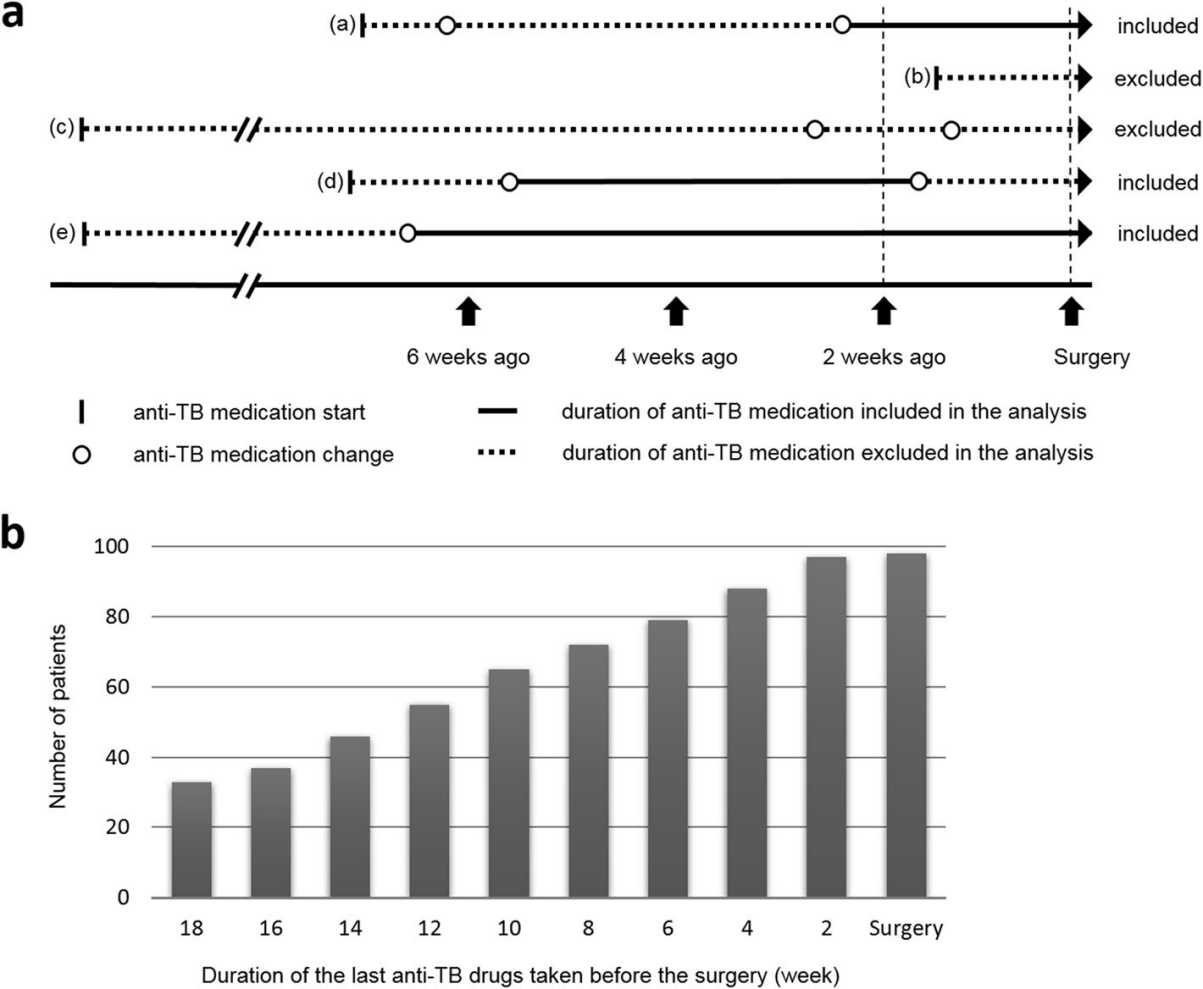
The duration of the last anti-TB drugs that were taken before the surgery. **a** Cases with a duration of more than 2 weeks for the last anti-TB treatment were included in the analysis. If the duration of the last anti-TB drug treatment was too short (within 2 weeks before the surgery), the type and duration of the anti-TB drugs taken just before that medication were calculated. **b** The period was divided into two-week intervals, and the number of patients included in each period was calculated. TB; tuberculosis

The relationship between anti-TB drugs and macrophage polarization was analyzed in the 98 patients of the MDR-TB/XDR-TB group. Long-term presurgical medication data were obtained. The treatment included 20 drugs. The most commonly used drugs were second-line oral agents, especially cycloserine, and amoxicillin/clavulanic acid (Additional file 1: Figure S1). The type, combination, and timing of the anti-TB drugs used were highly heterogeneous among the patients. Therefore, we estimated the duration of presurgical anti-TB drug treatment in each patient to select those who received the same anti-TB drugs continuously (Additional file 2: Figure S2). The duration of the last anti-TB drugs that were taken before the surgery was calculated. If the duration of the last anti-TB drug treatment was too short (within 2 weeks before the surgery), the type and duration of the anti-TB drugs taken just before that medication were determined (Fig. 3a). The period was divided into two-week intervals, and the number of patients included in each period is shown in Fig. 3b.

**Fig. 4:**
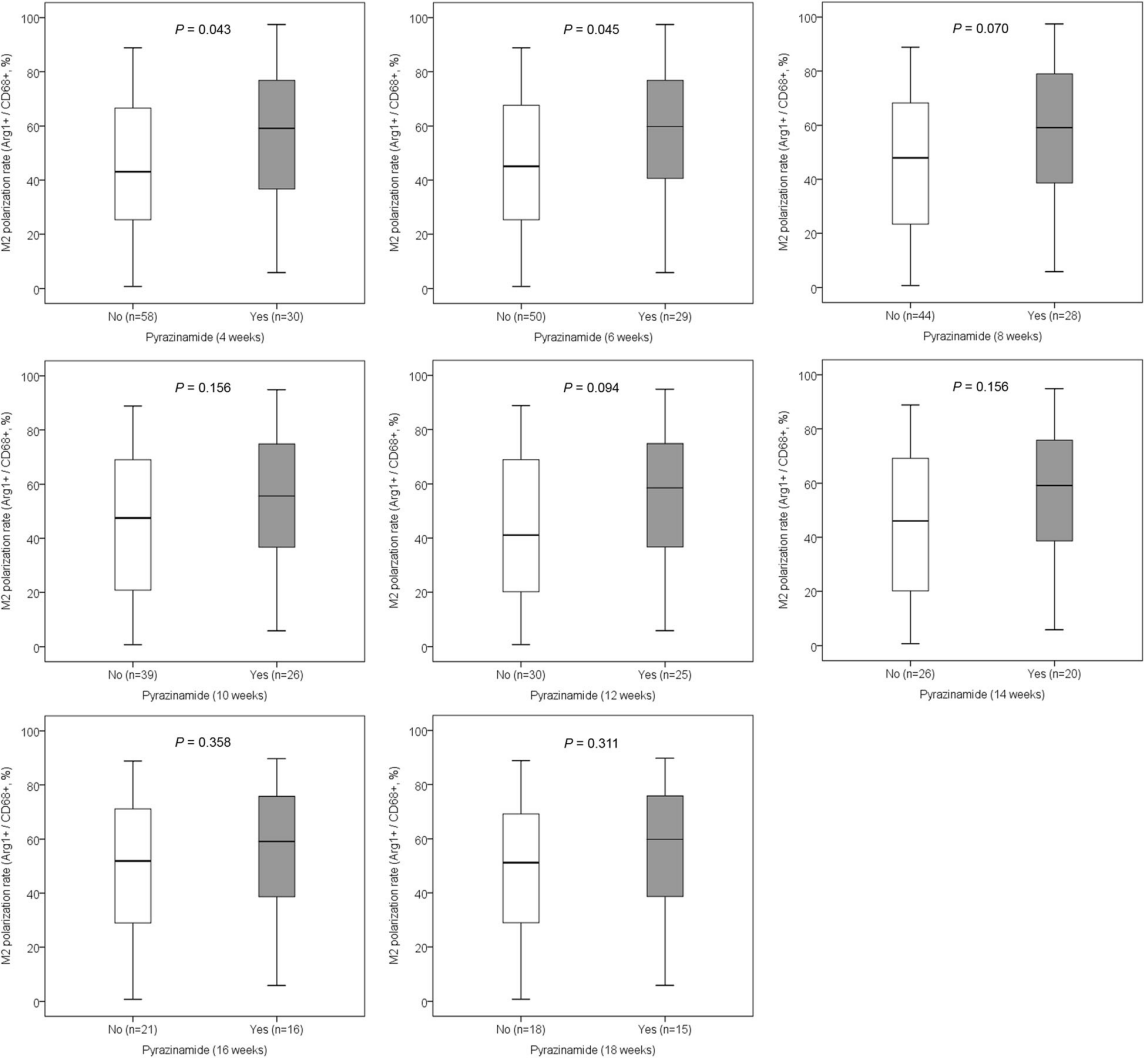
The association between the M2-like polarization rate and the duration using pyrazinamide. The M2-like polarization rate was significantly higher in patients who received anti-TB drugs containing pyrazinamide continuously for 4 or 6 weeks than in those who received anti-TB drugs not containing pyrazinamide. Arg1 = Arginase-1; TB = tuberculosis

**Fig. 5:**
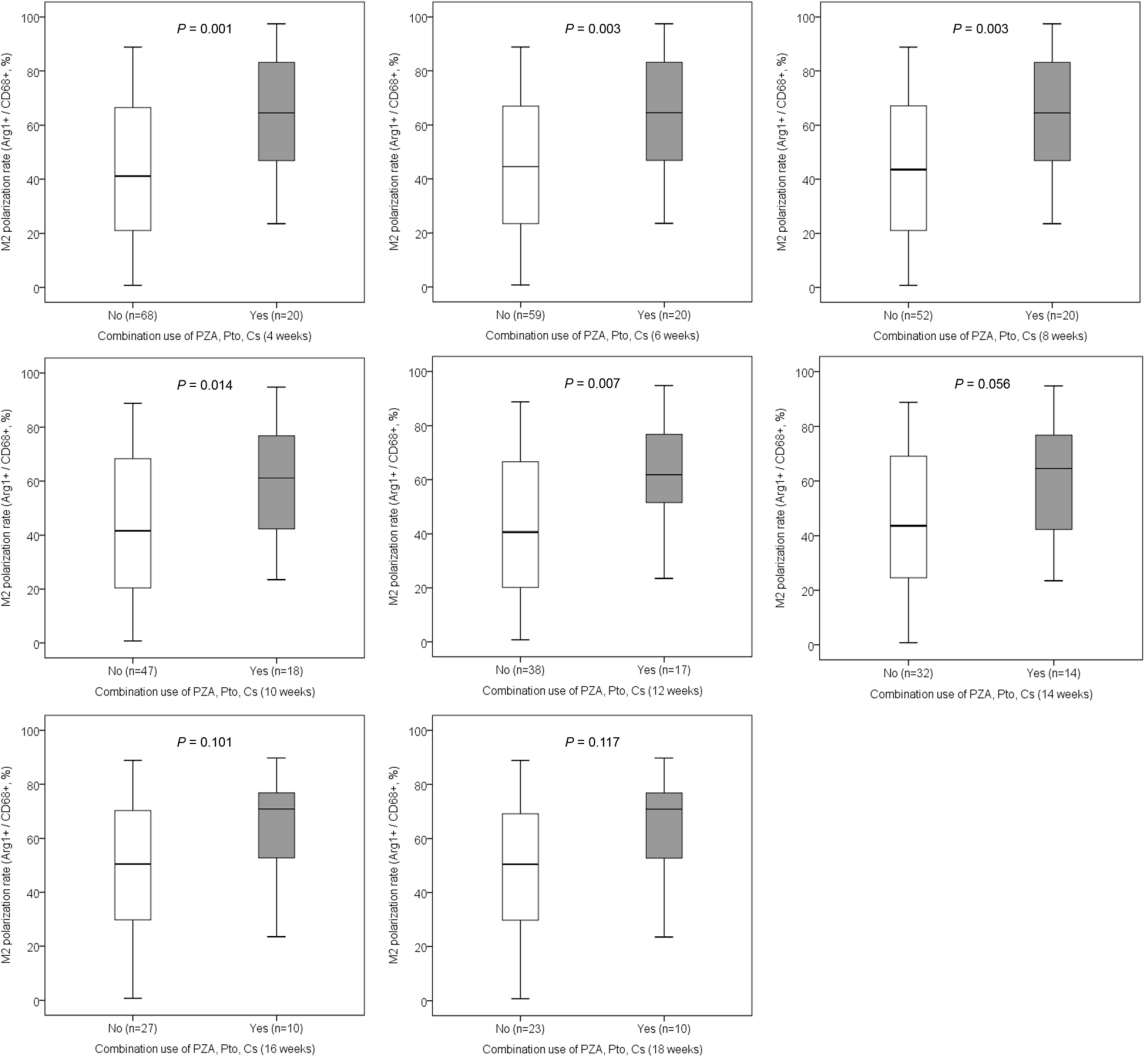
The association between the M2-like polarization rate and the duration using PZA, Pto and Cs. The use of anti-TB drug regimens including PZA, Pto and Cs for 4, 6, 8, 10, or 12 weeks was associated with a significantly higher M2-like polarization rate than was the use of anti-TB drug regimens without these three drugs. Arg1 = arginase-1; PZA = pyrazinamide; Pto = prothionamide; Cs = cycloserine; TB = tuberculosis

The association between the M1-like or M2-like polarization rate and the anti-TB drugs in each period was analyzed. The M1-like polarization rate was not associated with any anti-TB drug in any period. The M2-like polarization rate was significantly higher in patients who received anti-TB medications containing pyrazinamide continuously for 4 or 6 weeks compared with those who received anti-TB medications not containing pyrazinamide (Fig. 4). The use of any other drugs was not associated with the M2-like polarization rate. The use of anti-TB drug regimens including pyrazinamide, prothionamide, and cycloserine for 4, 6, 8, 10, or 12 weeks was associated with a significantly higher M2-like polarization rate than was the use of anti-TB drug regimens without these three drugs (Fig. 5). The M1-like polarization rate was not associated with any combination of anti-TB drugs in any period. There was no significant effect of age on the M2-like polarization rate in MDR-TB/XDR-TB patients (Additional file 3: Figure S3, Additional file 4: Figure S4, and Additional file 5: Figure S5).

## Discussion

Macrophages play an important role in the first-line immune defense against *Mtb* infection. Activated macrophages in a tissue microenvironment differentiate into two phenotypes. There is increasing evidence that M1- and/or M2 polarized macrophages are involved in the regression and/or progression of TB and have pro- and anti-inflammatory roles, respectively. Several recent reports have shown that macrophage polarization is involved in tuberculous granuloma formation [4, 5]. Our preliminary data showed that M2-like polarized macrophages were abundant in the lung tissues of MDR-TB patients [11]. In the current study, we compared the macrophage polarization rate in tuberculous granulomas between DS-TB and MDR-TB/XDR-TB patients and demonstrated that the M2-like polarization rate and the ratio of the M2-like polarization rate to the M1-like polarization rate were significantly higher in the MDR-TB/XDR-TB group than in the DS-TB group. The association between a high M2-like polarization rate and MDR-TB/XDR-TB was more pronounced in patients with a low M1-like polarization rate. Along with age, the M2-like polarization rate was also an independent associated factor in the multivariate analysis of MDR-TB/XDR-TB. Younger age as a risk factor for MDR-TB/XDR-TB has been reported in several studies [18, 19]. However, the M2-like polarization rate as an associated factor of MDR-TB/XDR-TB has never been reported before the present study. These findings agree with the pro- and anti-inflammatory processes that occur in the course of TB. The inhibition of the inflammatory response by low M1-like polarized macrophages and high M2-like polarized macrophages may affect the survival and acquisition of resistance of *Mtb* and the progression of tuberculosis [20–23]. Interestingly, the predominant M2-like polarized environment shifts back to an M1-like polarized environment after successful treatment of TB in *Mtb*-infected patients [24–27]. These observations suggest that anthropogenic regulation of the switch from M2-like polarized macrophages to M1-like polarized macrophages might control *Mtb* and TB.

The course of TB depends on the interaction among the host, *Mtb*, and drugs, in which each affects the other. The survival of *Mtb* against attack from macrophages is a key element in the progression of TB. An important mechanism in the survival of *Mtb* is the immunomodulation from an M1-like polarized environment to an M2-like polarized environment, and *Mtb* plays a key role in this immunomodulation [21, 26, 27]. However, the immunomodulation of the host may also be mediated by drugs. Especially in the case of TB, long-term medication with anti-TB drugs is necessary for successful treatment, and therefore, the effects of drug-mediated immunomodulation may be large. Some antimicrobial agents have immunomodulatory properties in vitro [12, 28], and some anti-TB drugs induce M2-like polarization of pleural macrophages in patients with pleuritic TB [16]. We observed a higher M2 macrophage polarization rate in TB patients treated with anti-TB drug regimens including pyrazinamide or a combination of pyrazinamide, prothionamide and cycloserine. Consistently, a previous report suggested that pyrazinamide treatment influences the host immune response [15, 16, 29]. These observations suggest that anti-TB drugs modulate the host immune response. Therefore, we postulate that long-term therapy for TB patients with anti-TB drugs associates with macrophage polarization by modulating cytokine and chemokine production.

Our study had some limitations. First is an inherent selection bias. This study was performed on patients who received surgical treatment, in order to analyze the properties of the tissue microenvironment. Only a small subset of all MDR-TB/XDR-TB patients underwent surgery, and most of them were patients whose disease progressed poorly. Therefore, these results should be only cautiously extrapolated to all MDR-TB/XDR-TB patients. In addition, our study cohort was a heterogeneous patient group classified based on the timing of medication and combinations of 20 anti-TB drugs used, which further decreased the number of patients included in the drug-polarization analysis. In this study, we used a single surface marker (iNOS for M1 and Arginase-1 for M2) to distinguish macrophage subtypes. In general, iNOS or Arginase-1 is considered a surface marker of M1 or M2 macrophages, respectively, but in some cases, both markers are expressed. Therefore, further verification using multiple surface markers for each macrophage subtype is required.

## Conclusions

To the best of our knowledge, this is the first report providing evidence of macrophage polarization in MDR-TB/XDR-TB. Our findings indicate that the M2-like polarization of macrophages is associated with MDR-TB/XDR-TB and anti-TB drug regimens including pyrazinamide or a combination of pyrazinamide, prothionamide and cycloserine. Further studies involving more patients and identifying the underlying mechanisms and causal relationships between M2 polarization of macrophages and anti-TB drugs in MDR-TB/XDR-TB will be needed.

## Supplementary information

**Additional file 1: Figure S1**. Anti-TB drugs used before surgery in the MDR-TB/XDR-TB groups.

**Additional file 2: Figure S2**. The schedule of anti-TB drugs in each patient of MDR-TB/XDR-TB groups before surgery.

**Additional file 3: Figure S3**. The difference of the M2-like polarization rate between the younger age group and the older age group in MDR-TB/XDR-TB patients.

**Additional file 4: Figure S4**. The association between the M2-like polarization rate and the duration using pyrazinamide in age subgroup.

**Additional file 5: Figure S5**. The association between the M2-like polarization rate and the duration using combination of PZA, Pto and Cs in age subgroup.

## Data Availability

The dataset used and/or analyzed during the current study is available from the corresponding author upon reasonable request.

## Abbreviations

CIs: Confidence intervals
DS-TB: Drug-susceptible tuberculosis
DSTs: Drug-sensitivity tests
IQR: Interquartile range
MDR-TB: Multidrug-resistant tuberculosis
MP ratio: Macrophage polarization ratio
*Mtb*: *Mycobacterium tuberculosis*
ORs: Odds ratios
TB: Tuberculosis
XDR-TB: Extensively drug-resistant tuberculosis
