# Supplementary material for: Different macrophage polarization between drug-susceptible and multidrug-resistant pulmonary tuberculosis
Source: BMC Infect Dis. 2020 Jan 29;20:81. doi: 10.1186/s12879-020-4802-9 (PMC6988333; doi:10.1186/s12879-020-4802-9)

**Figure S1**. **Anti-TB drugs used before surgery in the MDR-TB/XDR-TB groups**

The numbers are the cumulative number of patients using each drug for each week and the pink bars are the proportion of the 98 patients using each drug. MDR-TB = multidrug-resistant tuberculosis; XDR-TB = extensively drug-resistant tuberculosis; INH = isoniazid; RFP = rifampicin; EMB = ethambutol; PZA = pyrazinamide; RFB = rifabutin; Pto = prothionamide; Cs = cycloserine; PAS = para-aminosalicylic acid; Lfx = levofloxacin; Gfx = gatifloxacin; Mfx = moxifloxacin; Ofx = ofloxacin; Sm = streptomycin; Km = kanamycin; Cpm = capreomycin; Amk = amikacin; Evm = enviomycin; Amx/Clv = amoxicillin/clavulanic acid; Clr = clarithromycin; Lzd = linezolid.


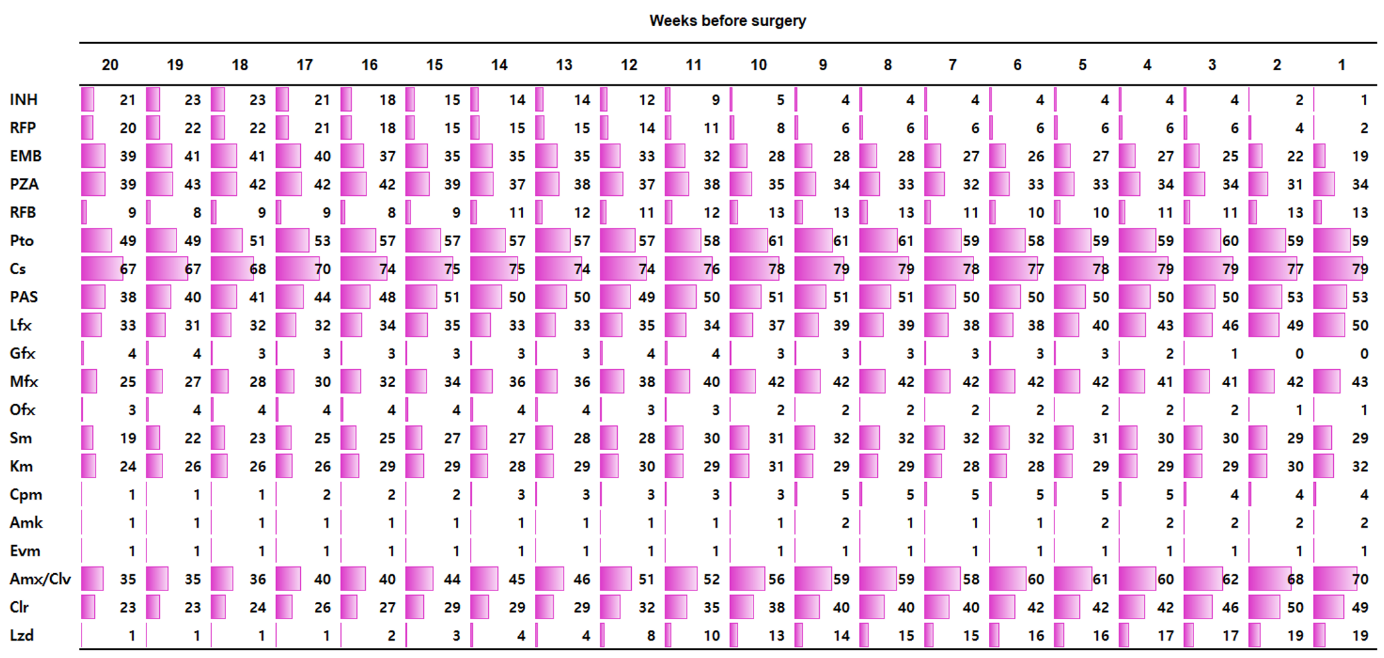

Supplement: Supplementary file 1 — Additional file 1: Figure S1. Anti-TB drugs used before surgery in the MDR-TB/XDR-TB groups. [file 12879_2020_4802_MOESM1_ESM.docx]
