# Supplementary material for: Different macrophage polarization between drug-susceptible and multidrug-resistant pulmonary tuberculosis
Source: BMC Infect Dis. 2020 Jan 29;20:81. doi: 10.1186/s12879-020-4802-9 (PMC6988333; doi:10.1186/s12879-020-4802-9)

**Figure S2**. **The schedule of anti-TB drugs in each patient of MDR-TB/XDR-TB groups before surgery**

The 20 drugs used in the 98 patients. Each color bar represents a drug used. MDR-TB = multidrug-resistant tuberculosis; XDR-TB = extensively drug-resistant tuberculosis; INH = isoniazid; RFP = rifampicin; EMB = ethambutol; PZA = pyrazinamide; RFB = rifabutin; Pto = prothionamide; Cs = cycloserine; PAS = para-aminosalicylic acid; Lfx = levofloxacin; Gfx = gatifloxacin; Mfx = moxifloxacin; Ofx = ofloxacin; Sm = streptomycin; Km = kanamycin; Cpm = capreomycin; Amk = amikacin; Evm = enviomycin; Amx/Clv = amoxicillin/clavulanic acid; Clr = clarithromycin; Lzd = linezolid.


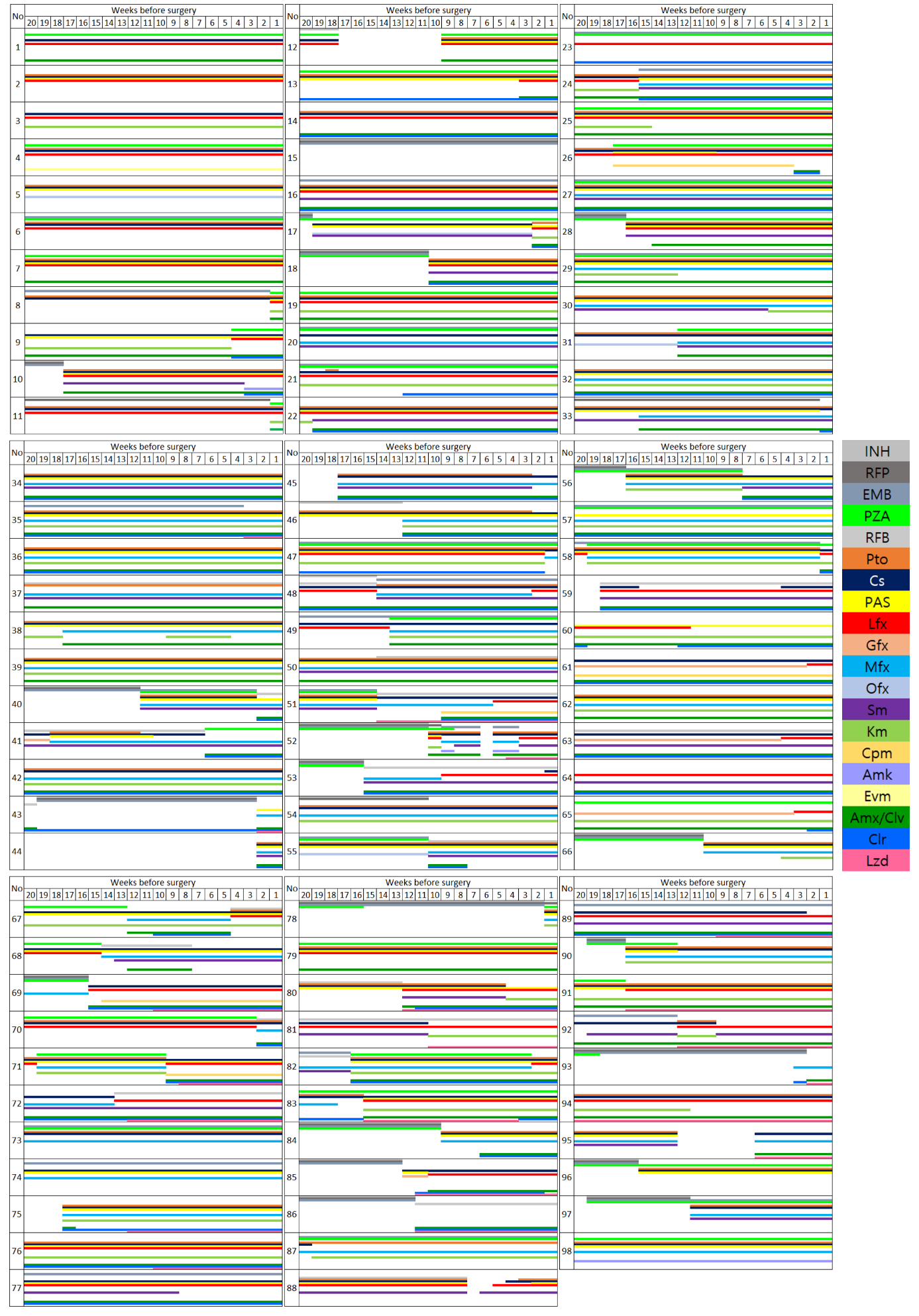

Supplement: Supplementary file 2 — Additional file 2: Figure S2. The schedule of anti-TB drugs in each patient of MDR-TB/XDR-TB groups before surgery. [file 12879_2020_4802_MOESM2_ESM.docx]
