# Supplementary material for: Different macrophage polarization between drug-susceptible and multidrug-resistant pulmonary tuberculosis
Source: BMC Infect Dis. 2020 Jan 29;20:81. doi: 10.1186/s12879-020-4802-9 (PMC6988333; doi:10.1186/s12879-020-4802-9)

**Figure S3**. **The difference of the M2-like polarization rate between the younger age group and the older age group in MDR-TB/XDR-TB patients.**

Subgroup of age was dichotomized by mean age (35.4 years old). The difference of the M2-like polarization rate between the younger age group (51.0 ± 26.6 %) and the older age group (43.5 ± 22.5 %) were not significant (*p*=0.138).


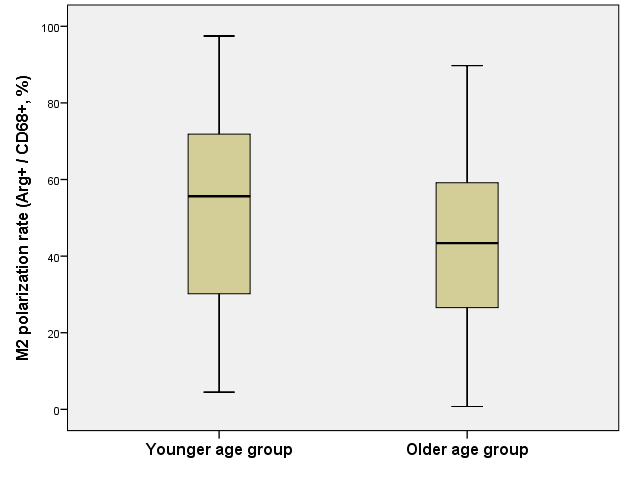

Supplement: Supplementary file 3 — Additional file 3: Figure S3. The difference of the M2-like polarization rate between the younger age group and the older age group in MDR-TB/XDR-TB patients. [file 12879_2020_4802_MOESM3_ESM.docx]
