# Supplementary material for: Different macrophage polarization between drug-susceptible and multidrug-resistant pulmonary tuberculosis
Source: BMC Infect Dis. 2020 Jan 29;20:81. doi: 10.1186/s12879-020-4802-9 (PMC6988333; doi:10.1186/s12879-020-4802-9)

**Figure S4**. **The association between the M2-like polarization rate and the duration using pyrazinamide in age subgroup.**

The M2-like polarization rate was significantly higher in patients who received the anti-TB drugs containing pyrazinamide compared with those who received the anti-TB drugs not containing pyrazinamide in younger age group. However, there was no significant differences in older age group. Arg1 = arginase-1; TB = tuberculosis.


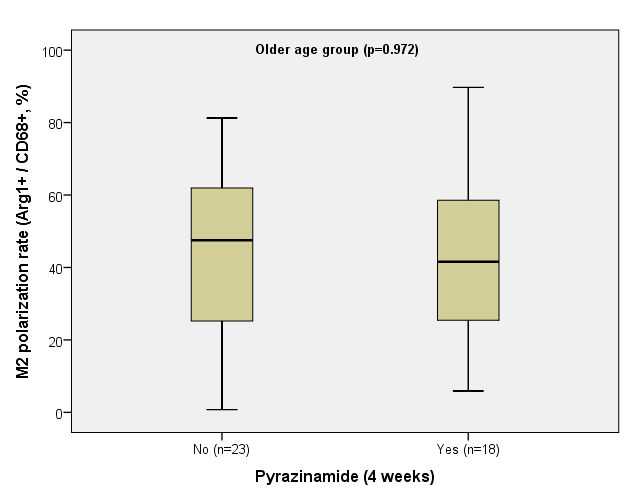

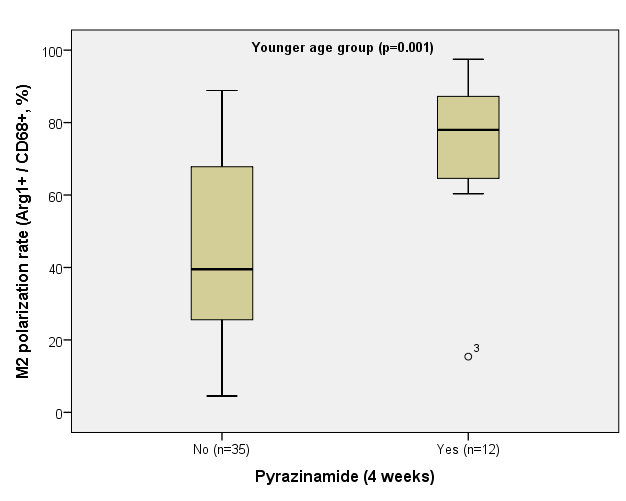


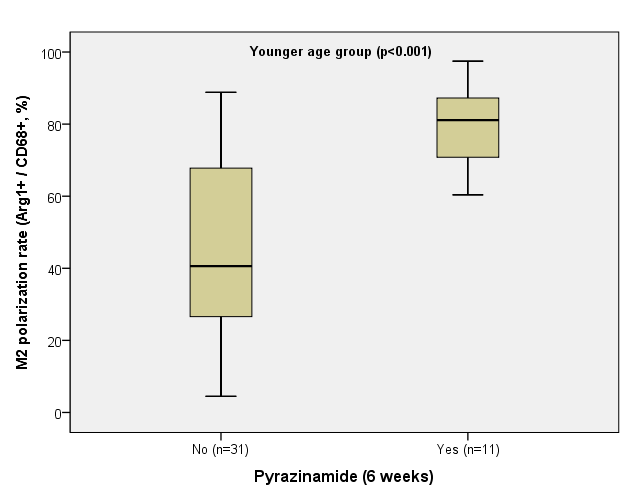


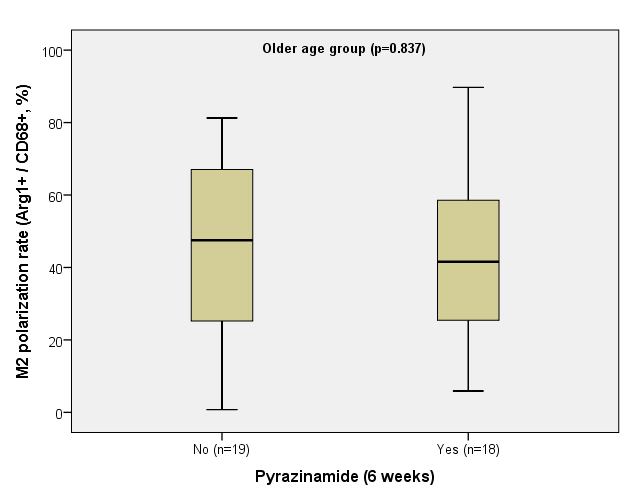


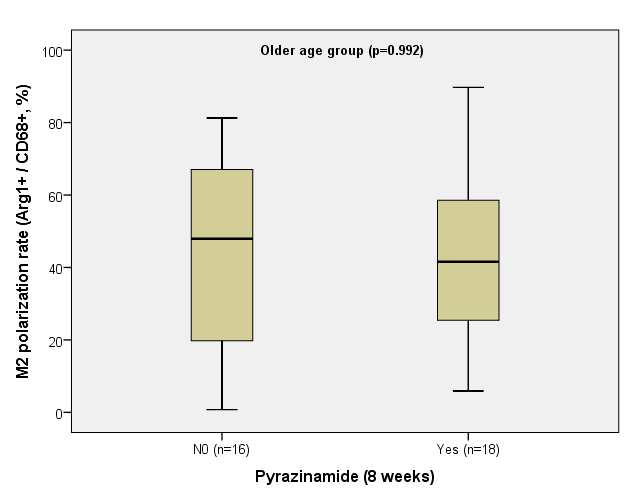

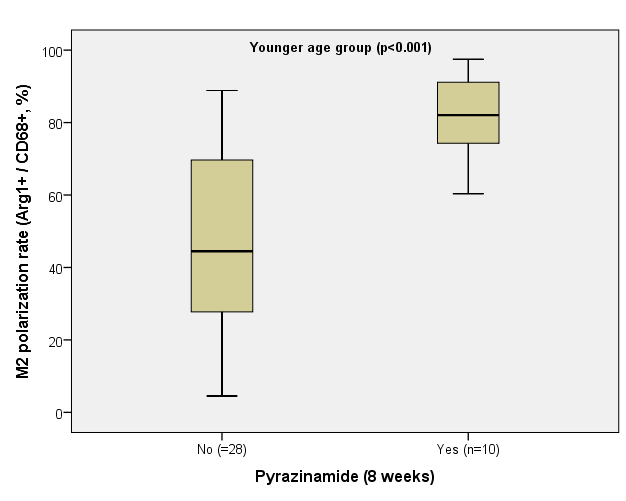


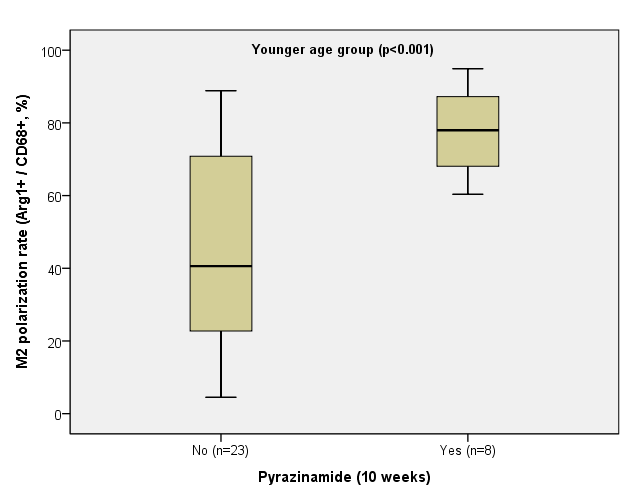


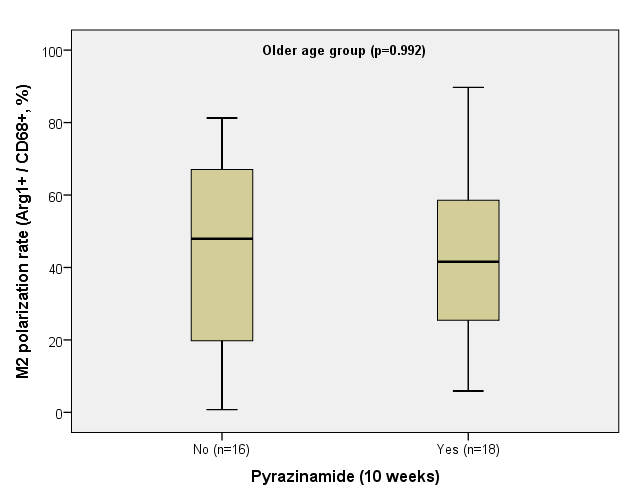


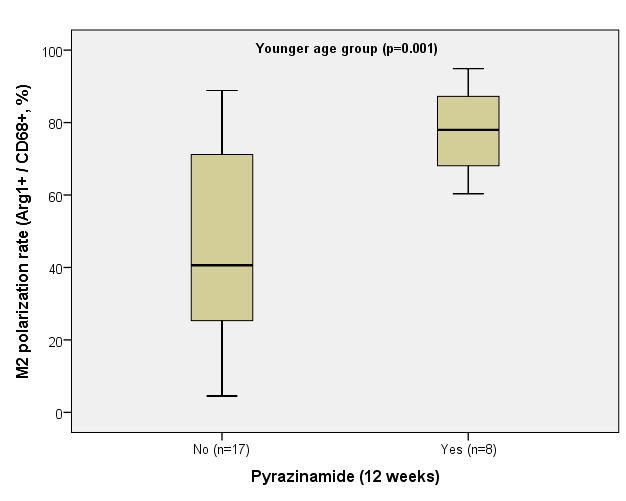


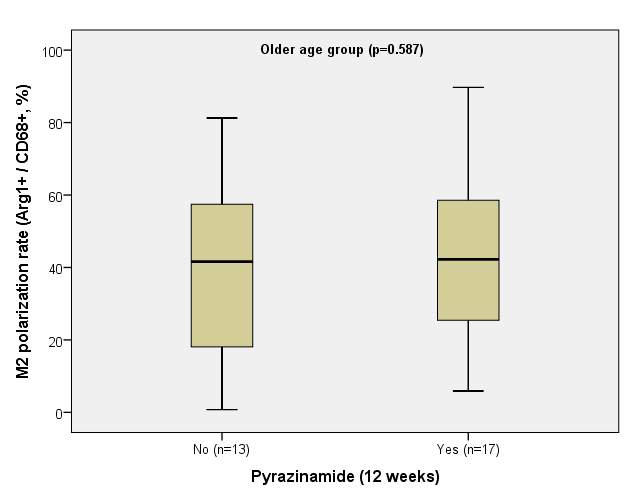

Supplement: Supplementary file 4 — Additional file 4: Figure S4. The association between the M2-like polarization rate and the duration using pyrazinamide in age subgroup. [file 12879_2020_4802_MOESM4_ESM.docx]
