# Supplementary material for: Different macrophage polarization between drug-susceptible and multidrug-resistant pulmonary tuberculosis
Source: BMC Infect Dis. 2020 Jan 29;20:81. doi: 10.1186/s12879-020-4802-9 (PMC6988333; doi:10.1186/s12879-020-4802-9)

**Figure S5**. **The association between the M2-like polarization rate and the duration using combination of PZA, Pto and Cs in age subgroup.**

The use of anti-TB drug regimens including PZA, Pto and Cs in younger age group was associated with a significantly higher M2-like polarization rate than the use of the anti-TB drug regimens without these three drugs. However, there was no significant differences in older age group. Arg1 = arginase-1; PZA = pyrazinamide; Pto = prothionamide; Cs = cycloserine; TB = tuberculosis.


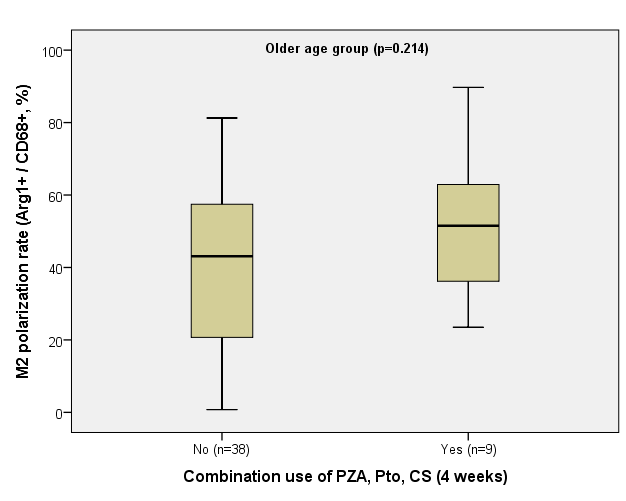

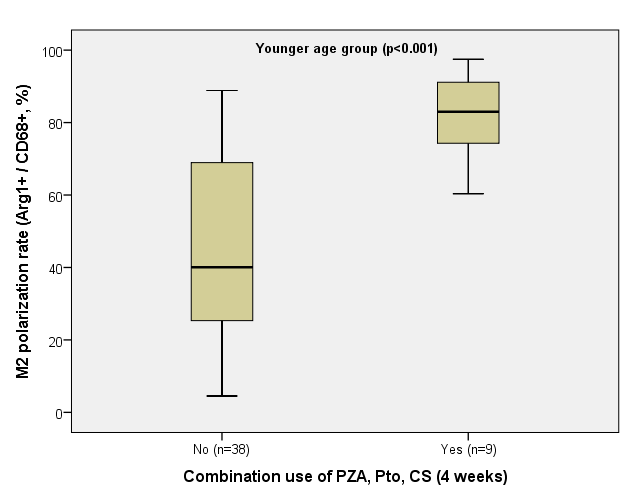


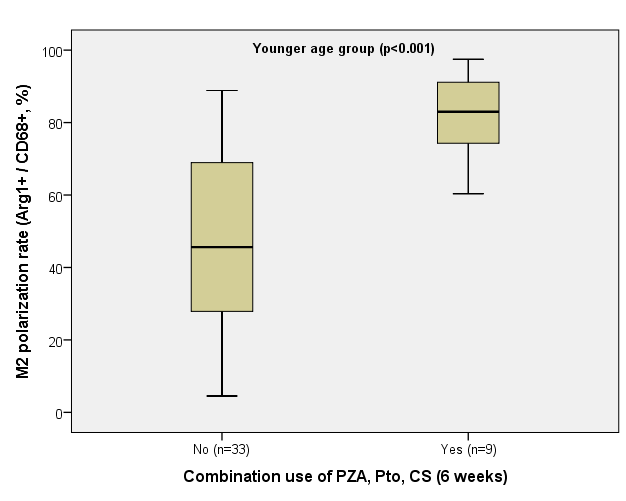

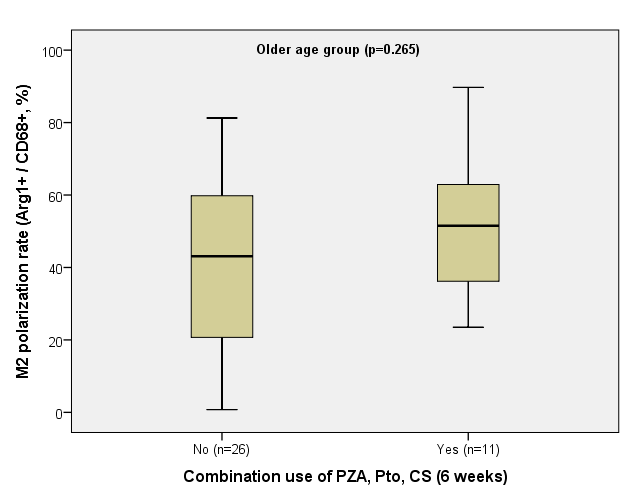


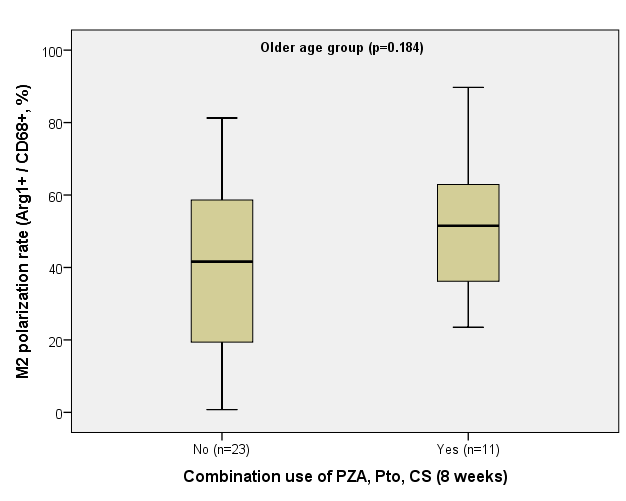

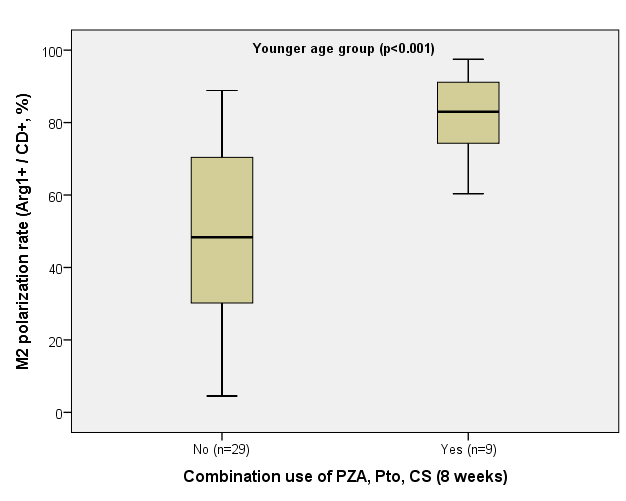


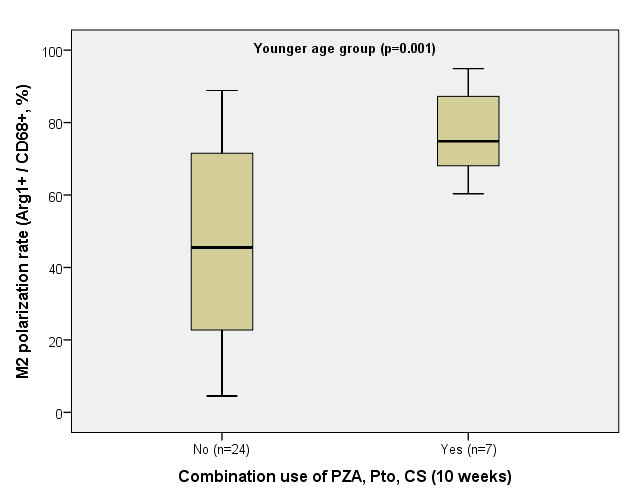


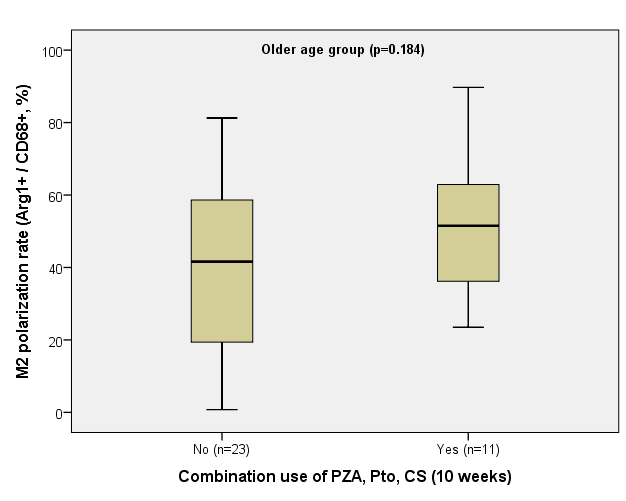


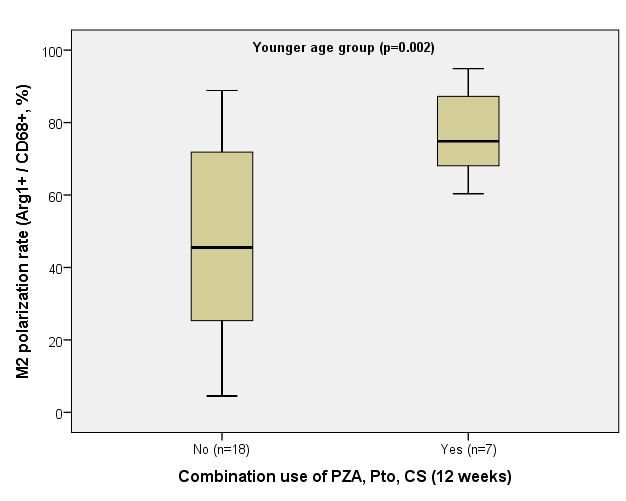


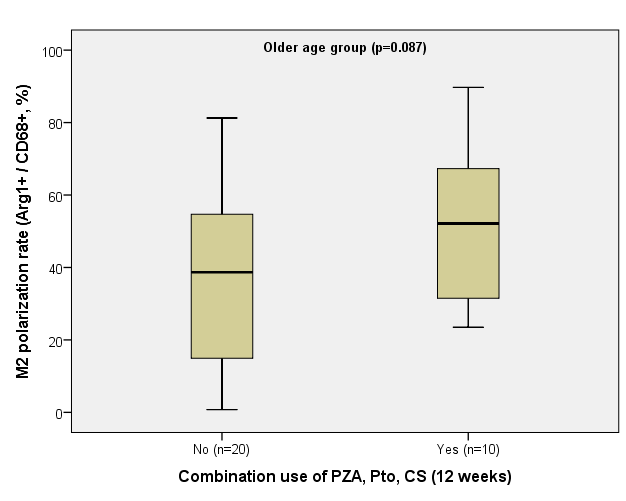

Supplement: Supplementary file 5 — Additional file 5: Figure S5. The association between the M2-like polarization rate and the duration using combination of PZA, Pto and Cs in age subgroup. [file 12879_2020_4802_MOESM5_ESM.docx]
